# Complete mitogenome of asiatic lion resolves phylogenetic status within *Panthera*

**DOI:** 10.1186/1471-2164-14-572

**Published:** 2013-08-23

**Authors:** Snehal B Bagatharia, Madhvi N Joshi, Rohan V Pandya, Aanal S Pandit, Riddhi P Patel, Shivangi M Desai, Anu Sharma, Omkar Panchal, Falguni P Jasmani, Akshay K Saxena

**Affiliations:** 1 Gujarat State Biotechnology Mission, Department of Science and Technology, Government of Gujarat, Block-11, 9th Floor, Udyog Bhavan, Sector 11, Gandhinagar 382 017, Gujarat, India

**Keywords:** Asiatic lion, Big cats, Panthera leo persica, Mitogenome, Ion torrent, Phylogeny, Evolution, Felidae, Divergence time

## Abstract

**Background:**

The origin, evolution and speciation of the lion, has been subject of interest, debate and study. The present surviving lions of the genus *Panthera* comprise of eight sub-species inclusive of Asiatic lion Panthera leo persica of India's Gir forest. Except for the Asiatic lion, the other seven subspecies are found in different parts of Africa. There have been different opinions regarding the phylogenetic status of *Panthera leo*, as well as classifying lions of different geographic regions into subspecies and races. In the present study, mitogenome sequence of *P*. *leo persica* deduced, using Ion Torrent PGM to assess phylogeny and evolution which may play an increasingly important role in conservation biology.

**Results:**

The mtDNA sequence of *P*. *leo persica* is 17,057 bp in length with 40.8% GC content. Annotation of mitogenome revealed total 37 genes, including 13 protein coding, 2 rRNA and 22 tRNA. Phylogenetic analysis based on whole mitogenome, suggests *Panthera pardus* as a neighbouring species to *P*. *leo* with species divergence at ~2.96 mya.

**Conclusion:**

This work presents first report on complete mitogenome of *Panthera leo persica*. It sheds light on the phylogenetic and evolutionary status within and across Felidae members. The result compared and evaluated with earlier reports of Felidae shows alteration of phylogenetic status and species evolution. This study may provide information on genetic diversity and population stability.

## Background

Five big charismatic cats: *Panthera leo* (lion), *Panthera tigris* (tiger), *Panthera onca* (jaguar), *Panthera pardus* (leopard) and *Uncia uncia* (snow leopard) have been placed taxonomically in Pantherinae subfamily. They have been drawing attention of biologists due to important ecological roles [1]. Consequently, extensive information is available on their natural history, morphology, behaviour, reproduction, evolutionary history and population genetic structure, which provides a rich basis for interpreting genetic data [2]. The information is still not adequate to overcome their highly threatened status. Hence, molecular study is vital to further explore the genetic information which can be helpful for conservation.

*Panthera leo* has two geographically isolated populations; *Panthera leo leo* (African lion), and *Panthera leo persica* (Asiatic lion) [3]. The Asiatic lion population is accorded endangered species status under the Indian Wildlife Protection Act, consisting only 411 wild animals [4]. This population exist in and around Gir forest in the southwest part of Saurashtra region in the State of Gujarat, India. Presence of geographically confined single population having its origin from small nucleus group and constituting single gene pool, raises concerns about genetic diversity in Asiatic lion population. Morphological and molecular approaches like allozyme study [5] microsatellite analysis [6], protein markers and mitochondrial 12S gene [7] have been used to unveil the evolutionary history of this species. Earlier efforts have been made to undertake the population study of *Panthera leo persica* (NCBI Taxonomy ID: 83386) and other allied species [8] to establish phylogenetic status. However, it has been found to be perplexing [7].

For the last decade, mitochondrial DNA has been one of the most commonly used molecular marker in vertebrates for studying phylogeny and evolutionary relationships [9] among closely related species and subspecies [10]. It can reveal evolutionary relatedness and elucidate large numbers of genome-level characteristics, such as relative arrangements of genes [11]. It also has a great importance for the molecular identification of species. Cytochrome b and Cytochrome oxidase subunit I [12] are mitochondrial genes widely used for molecular identification of animals. Further, key mitochondrial features - a lack of recombination, essentially maternal inheritance, high evolutionary rate, compact size, and conserved gene order [9], have led to its wide spread use.

Earlier, phylogenetic relationship of the genus *Panthera* was studied using morphological, biochemical as well as molecular characters but it is still debatable and troublesome because of large disparities between these studies. The difficulty in resolving their phylogenetic relationships is a result of (i) a poor fossil record, (ii) recent and rapid radiation during the Pliocene, (iii) individual speciation events occurring within less than 1 million years (iv) probable introgression between lineages following their divergence [13]. Phylogenetic relationship and position of *Panthera leo*, amongst the genus *Panthera*, has some knowledge gaps. The present study aims to provide some insights through the complete mitogenome of the Asiatic lion.

## Results and discussion

Mitochondrial genome sequence of any subspecies of *Panthera leo*, is not reported in literature. Present study describes sequencing of complete mitogenome of Asiatic Lion (*P*. *leo persica*) with the use of next generation sequencing technology on Ion Torrent^PGM^ platform. The relationship of lion subspecies with other Felidae species and their evolutionary status has also been described on the basis of comparative analysis of mitogenomes.

### Base composition of mitochondrial genome of *Panthera leo persica*

The complete mitogenome sequence of *P*. *leo persica* is 17,057 bp in length (GenBank accession No: KC834784), which is larger than *Panthera pardus* (16,964) and *Uncia uncia* (16,773). The base composition of mitogenome of *P*. *leo persica* is A, 5445 bp (31.92%); T, 4650 bp (27.26%); C, 4492 bp (26.33%); G, 2470 bp (14.48%); A+T 9939 bp (59.18%), G+C 6879 (40.81%). Base A is highest among the 4 bases; G is the lowest. Features of lion mitogenome were compared with genomes of Felidae family. The genome size varies from 16773 bp to 17153 bp mainly because of variation in control region length. GC content of all the genomes is ranging from 39.37% to 41.19% (Table 1).

**Table 1:** General mitogenome features of Felidae family

**Species name**	**Common name**	**NCBI Taxonomy ID**	**Accession no.**	**CR Length**	**GC %**	**Genome length**
*Acinonyx jubatus*	Cheetah	32536	NC_005212	1602	39.37	17047 bp
*Prionailurus bengalensis euptilurus*	Amur leopard cat	300877	NC_016189	1547	39.55	16990 bp
*Puma concolor*	Puma	9696	NC_016470	1706	39.78	17153 bp
*Felis catus*	Domestic cat	9685	NC_001700	1559	40.33	17009 bp
*Panthera leo persica*	Asiatic lion	83386	KC834784	1603	40.8	17057 bp
*Uncia uncia*	Snow leopard	29064	NC_010638	1318	40.97	16773 bp
*Lynx rufus*	Bobcat	61384	NC_014456	1606	41.01	17056 bp
*Panthera pardus*	Leopard	9691	NC_010641	1514	41.11	16964 bp
*Panthera tigris*	Tiger	9694	NC_010642	1537	41.14	16990 bp
*Neofelis nebulosa*	Clouded leopard	61452	NC_008450	1400	41.15	16844 bp
*Panthera tigris amoyensis*	Amoy tiger	253258	NC_014770	1550	41.19	17001 bp

### Structure of mitogenome of *Panthera leo persica*

**Figure 1:**
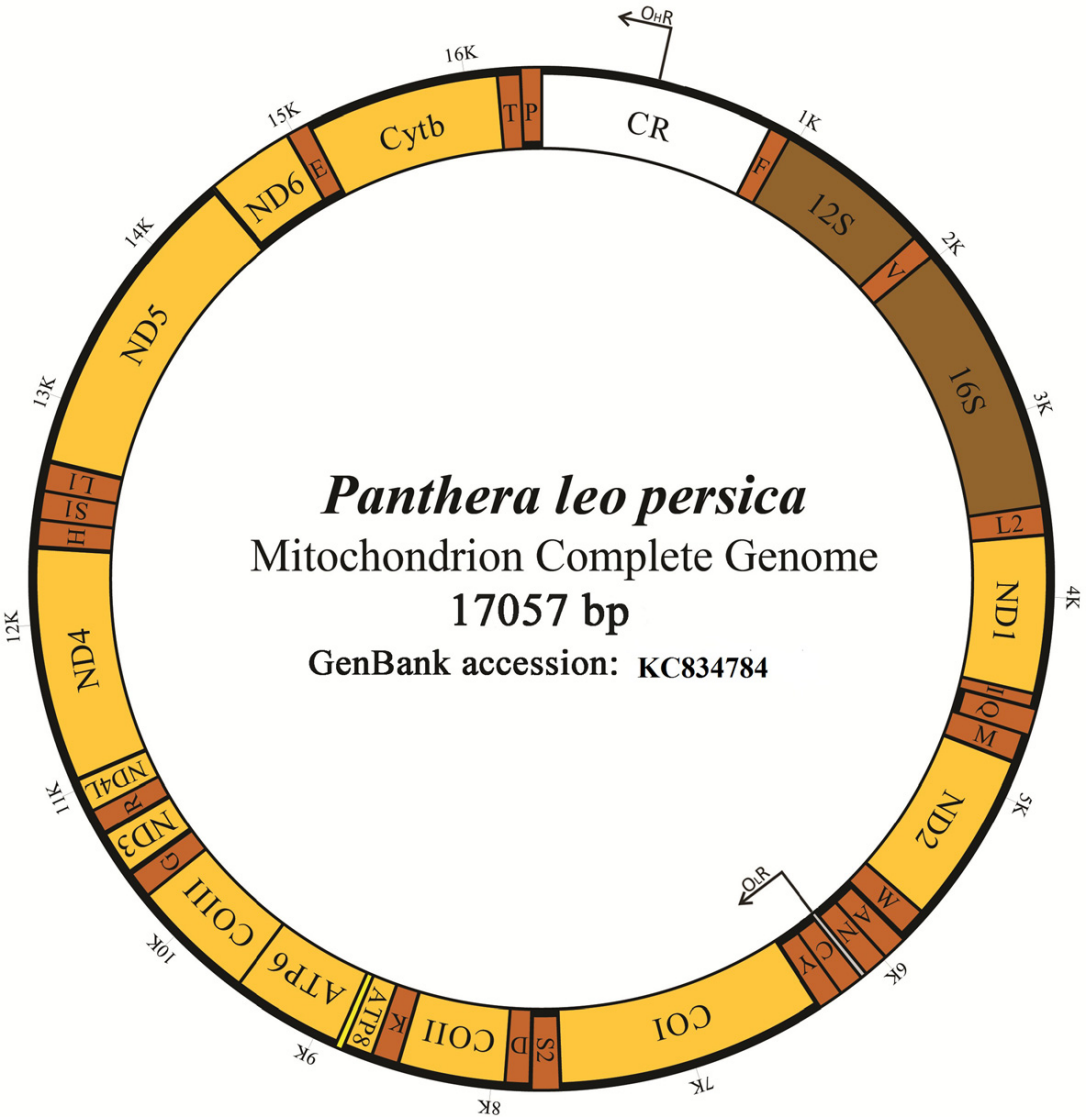
**Complete mitochondrial genome organization of *Panthera leo persica*.**

Location of the mitochondrial genes were deduced, using online annotation server MITOS [14]. Complete mitogenome contained 37 genes which include 13 protein coding genes, 2 rRNA genes, 22 tRNA genes and the control region (Figure 1). Gene order and origin of reading frame of all protein coding genes were identical to Carnivora. Major difference in *P*. *leo persica* mitogenome was observed in size of the control region (1,603 bp).

Transfer RNA was annotated in a single letter amino acid code. OHR and OLR represent the replication origins of H strand and L strand. Except for ND6, all protein coding genes were H strand encoded. Position of genes and control regions (CR) were indicated with Arabic numerals (Table 2).

**Table 2:** **Location of features in the mitochondrial DNA of *Panthera leo persica***

**Name**	**Position**		**Codon**		**Chain**	**Length**
	**Start**	**Stop**	**Start**	**Stop**		
trnF(gaa)	868	939			H	72
rrnS	940	1903			H	964
trnV(tac)	1902	1970			H	69
rrnL	1969	3544			H	1576
trnL2(taa)	3545	3619			H	75
nad1	3622	4572	ATG	TAA	H	951
trnI(gat)	4578	4646			H	69
trnQ(ttg)	4644	4717			L	74
trnM(cat)	4719	4787			H	69
nad2	4782	5819	ATA	TAG	H	1038
trnW(tca)	5830	5898			H	69
trnA(tgc)	5914	5982			L	69
trnN(gtt)	5984	6056			L	73
trnC(gca)	6090	6154			L	65
trnY(gta)	6155	6220			L	66
cox1	6213	7754	ATT	TAA	H	1542
trnS2(tga)	7764	7832			L	69
trnD(gtc)	7839	7907			H	69
cox2	7908	8588	ATG	TAA	H	681
trnK(ttt)	8595	8662			H	68
atp8	8664	8861	ATG	TAA	H	198
atp6	8825	9499	ATG	TAA	H	675
cox3	9505	10287	ATG	TAG	H	783
trnG(tcc)	10289	10357			H	69
nad3	10343	10702	ATC	TAG	H	360
trnR(tcg)	10705	10773			H	69
nad4l	10774	11067	ATG	TAA	H	294
nad4	11064	12431	ATG	TA	H	1368
trnH(gtg)	12442	12510			H	69
trnS1(gct)	12511	12569			H	59
trnL1(tag)	12570	12639			H	70
nad5	12631	14445	ATA	TAA	H	1815
nad6	14450	14974	ATT	TAA	L	525
trnE(ttc)	14972	15040			L	69
Cob	15044	16177	ATG	AGA	H	1134
trnT(tgt)	16184	16253			H	70
trnP(tgg)	16254	16320			L	67

### Proteins and codons

The studied mitogenome comprise of 13 protein coding genes (11,364 bp) and share 66.62% of total genome. These genes encode 3788 amino acids (Table 2). The longest gene was ND5 (1815 bp) and the shortest gene was ATP8 (198 bp). The start and termination codons appeared universal among species. ATG was commonly used as start codon for all the genes except ND2, ND5, COX1, ND6 and ND3, which used ATA, ATT and ATC, respectively. TAA was commonly found as stop codon while Cyt b ended with AGA. Termination of Cyt b gene with AGA, is typical for mammalian species [15-17] which is distinct from others such as amphibians [18], reptiles [19,20], Aves [21] and Nematoda [22]. ND3, ND4, ND2 and COX3 have incomplete stop codons. These incomplete termination codons are common in metazoan mitogenome and can be converted into complete ones by poly A after transcription (TAA) [23].

### tRNA and rRNA

**Figure 2:**
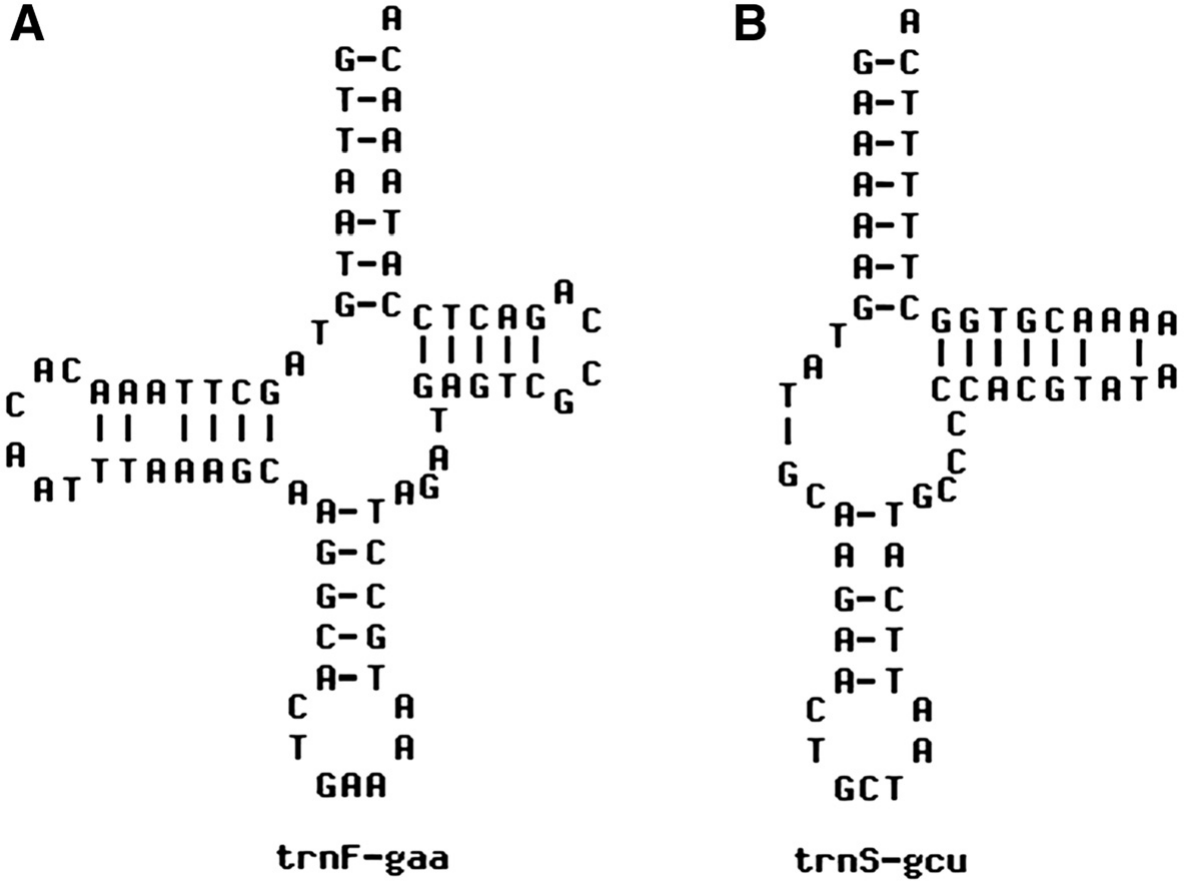
**Secondary structures of tRNAs.** The secondary structure of **(A)** - tRNA^phe^. **(B)** - tRNA^ser^.

Total 22 tRNA genes were found in mitogenome of *P*. *leo persica*, amongst these tRNA^ser^ and tRNA^leu^ had two termination codons (Table 2). Length of the tRNA genes in vertebrates is reported from 59 to 75 bp [24]. In this study, the shortest tRNA is tRNA^ser^ (59 bp) and longest is tRNA^leu^ (75 bp). Cloverleaf secondary structure of tRNAs of this genome was predicted, out of which representative structures of tRNA^phe^ and tRNA^ser^ are shown (Figure 2). Out of all tRNAs, tRNA^ser^ lacked the “DHU” arm [25]. The location of 12S rRNA (964 bp) was between tRNA^phe^ and tRNA^val^ and 16S rRNA (1576 bp) was between tRNA^val^ and tRNA^leu^.

### Control region

**Figure 3:**
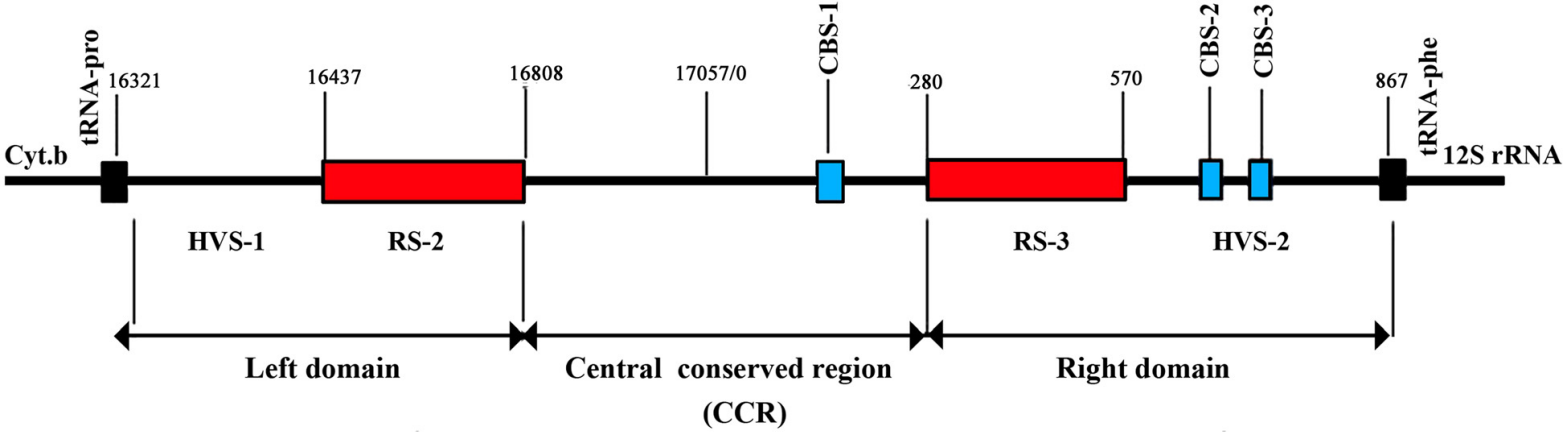
**Structure of control region of *Panthera leo persica *mitogenome.** Showing conserved blocks, location of repetitive sequences (RS), and other defined domains.

The control region of the mitogenome of *P*. *leo persica* is located between tRNA^Pro^ and tRNA^Phe^. It contains only promoters and regulatory sequences for replication and transcription, but no structural genes (Figure 3). CR is divided into three parts: the left domain (16,321-16,808bp) which includes the hyper variable segment HVS-1, the central conserved region (CCR) of 529 bp, and the right domain of (280–867 bp), which include HVS-2. Further the repetitive sequence RS-2 is in the left domain located at the 5’ end. It consists of long repetitive motif of about 80 bp which is repeated 4.7 times (3’-CCCCATGAATATTAAGCATGTACAGTAGTTTATATATATTACATAAGGCATACTATGTATATCGTGCATTAACTGCTTGT–5’). RS-3 is located in right domain at 3’ end. It consists of short repeat units of 14 bp which is repeated 20 times (3’-ACACGTACACACGT-5’). RS-3 is highly variable in the arrangement of specific motifs and is heteroplasmic. It is varied in terms of repeat numbers and motif among Felidae species (Table 3) [26]. When there are repeats in the genome longer than the read length, it is important to confirm NGS data with capillary sequencing. Here, RS-2 and RS-3 repeats have longer region than read length of Ion Torrent PGM. Hence RS-2 and RS-3 were validated with capillary sequencing and capillary data (RS2 and RS3 repeats) was replaced in original genome obtained from NGS. Conserved sequence blocks (CSB-2 and CSB-3) are in HVS-2. CSB-1 is in the CCR, which is located between RS-2 and RS-3. CR of *P*. *leo persica* comprises 1,603 bp and it contains origin of heavy chain replication. The capillary data of RS2 and RS3 repeats was submitted to TRA (Trace archive Id: TR-16480).

**Table 3:** Repeat sequence comparison in Felidae family

**Species Name**	**RS2 (No. of repeats)**	**RS3 (No. of repeats)**	**RS3 motif length**	**RS3 motif**	**Genome length**
*Panthera leo persica*	4.7	21.1	14	ACACGTACACACGT	17057 bp
*Panthera pardus*	1.9	48.5	8	ACACGTAC	16964 bp
*Uncia uncial*	2	12.4	14	TACACGTACACGTA	16773 bp
*Panthera tigris*	2	47.4	8	CACGTATA	16990 bp
*Panthera tigris amoyensis*	2.1	47.5	8	ACACGTAC	17001 bp
*Neofelis nebulosa*	Not found	37.7	6	ACACGT	16844 bp
*Felis catus*	3.2	36.6	8	ACACGTAC	17009 bp
*Prionailurus bengalensis euptilurus*	3.1	37.4	8	CACGTATA	16990 bp
*Lynx rufus*	3.8	43.5	8	ACACGTAC	17056 bp
*Acinonyx jubatus*	3.5	50.2	6	CGTACA	17047 bp
*Puma concolor*	4.7	45.6	8	TACACGTA	17153 bp

RS2 motif sequence remains conserved, while RS3 motif varies in Felidae family.

### Phylogenetic analyses based on gene cluster

**Figure 4:**
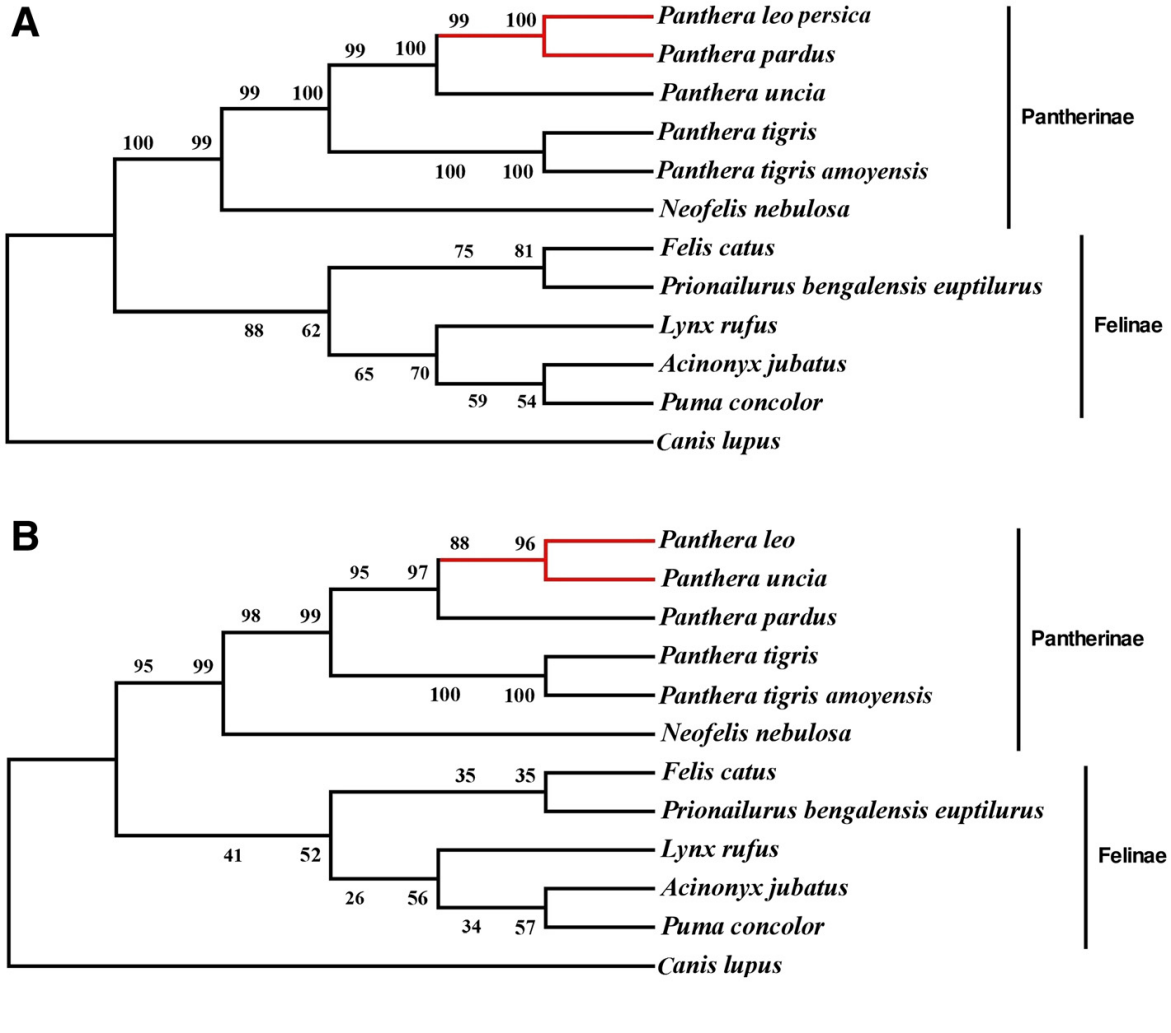
**Maximum parsimony and Maximum likelihood based gene cluster phylogeny. (A)** Full gene sequences and **(B)** partial gene sequences. Numbers represent bootstrap values.

Phylogenetic analyses was carried out on the basis of partial [27] and full gene sequences of 12S rRNA, 16S rRNA, ND2, ND4, ND5, ATP8 and Cyt b using ML, MP methods (Figure 4). The monophyly of the genus *Panthera*, including *P*. *leo persica*, *Uncia uncia*, *Panthera pardus*, *Panthera tigris amoyensis*, *Panthera tigris*, and *Neofelis nebulosa* was clearly depicted. Results demonstrate 100% bootstrap value in ML method and 99% in MP method, using full gene sequences of *P*. *leo persica* (Figure 4A). Previously sequenced *Panthera leo* partial genes formed node with *Uncia uncia* with 96% bootstrap value in ML method and 88% in MP method (Figure 4B). This study strongly supports that *P*. *leo persica* is a sister species of *P*. *pardus*, than of *U*. *uncia*, as earlier reported [27]. *N*. *nebulosa* was forming distinct branch. *P*. *tigris* and *P*. *tigris amoyensis* being same species, formed different branch in the Panthera node while *U*. *uncia* was placed in the lineage between tiger and lion-leopard node.

### Estimates of divergence times

**Figure 5:**
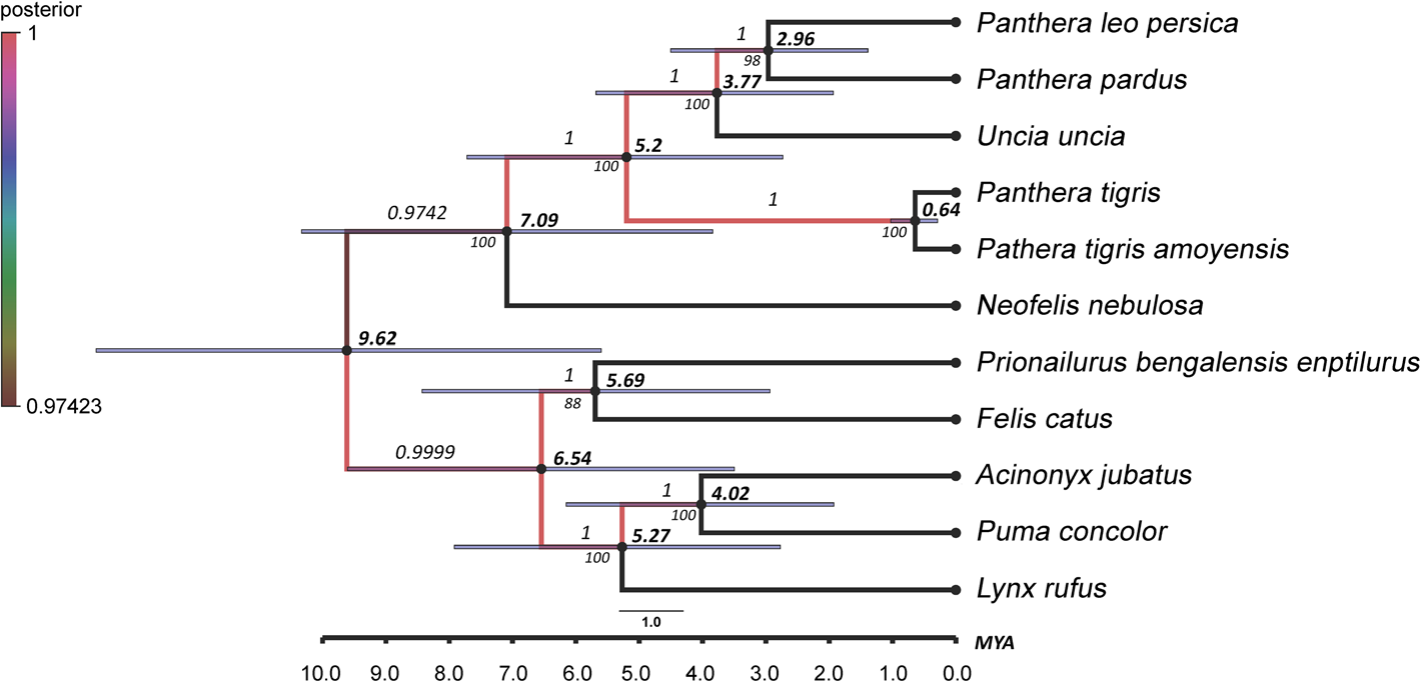
**Phylogenetic tree and divergence time estimates.** ML and Bayesian analysis based on whole mitogenomes. Numbers above the nodes represent posterior probabilities and numbers below the nodes are bootstrap values. Bold numbers represent estimated divergence times. The 95% highest posterior density estimates for each clade are presented by bars.

The ML and Bayesian analyses based on 11 complete mitochondrial genomes, excluding control region yielded the identical tree topology supported by high bootstrap value (above 90%, Figure 5) and high posterior probabilities (above 0.97, Figure 5). Uncorrelated lognormal relaxed clock (Figure 5) and strict molecular clock model (Additional file 1: Figure S1) were studied to estimate divergence times. Data of uncorrelated lognormal relaxed clock were considered for lineage specific rate heterogeneity. Divergence time was estimated using single calibration point at 10.78 mya for Felidae individuals, assuming common ancestor between genus *Panthera* and other Felinae species [28,29]. BEAST analysis suggested divergence time of Felidae individual was 9.62 mya (Figure 5) from the common ancestor. The divergence of *P*. *leo persica* was estimated 2.96 mya from the closely related species *P*. *pardus* (ML, 98%; Bayesian, 1.00). Divergence of other individuals; *N*. *nebulosa* (7.09 mya), *P*. *tigris* (5.2 mya) and *U*. *uncia* (3.77 mya). Estimation of divergence times of genus *Panthera* in the present study reconfirms earlier reports [30,31].

## Conclusion

Present study is the first report of complete mitogenome of *P*. *leo persica* among all eight subspecies. The evolutionary timeline estimates of big cats based on mitogenome analysis, indicates existence of common ancestor of *P*. *leo* and *P*. *pardus* 2.96 mya in modern Piacenzian era. The significant degree of genetic differentiation of Asiatic lion from other big cats, suggest independent evolution of subspecies described as *P*. *leo persica*. Complete mitogenome sequence provide sufficient information to resolve the events of divergence of Felidae.

## Materials and methods

### Sample collection and DNA extraction

Tissue sample of a freshly dead lion cub (female, 1.5 yrs) with chip ID 00-06B7-22E3 was collected from Sakkarbag Zoo, Junagadh, Gujarat, India (N 21.540848, E 70.468481). The samples were immediately transferred to laboratory and stored in Allprotect Tissue Reagent (Qiagen) at −80°C. Tissue sample was homogenized in liquid nitrogen and DNA extraction was carried out using BioVison Mitochondrial DNA Isolation Kit (BioVision Research Products, CA, USA) following manufacturer’s protocol.

### Sequencing

DNA quality such as ratio of absorbance at 260/280 nm and 260/230 nm was measured using Nanophotometer (Imlpen). Qubit® 2.0 Fluorometer was used to obtain an accurate quantitation of DNA. Library was prepared using Ion Express Plus Fragment library kit (Life Technologies). Mitochondrial DNA was sheared into blunt ended fragments by enzymatic lysis using Ion Shear Plus Reagents (Life Technologies). The fragmented DNA was ligated to Ion-compatible adapters, followed by nick repair to complete the linkage between adapters and DNA inserts. Sequencing was performed using Ion Express Template 300 chemistry (Life Technologies) on 316 chip following manufacturer’s protocol.

Capillary Sequencing of D-loop region was performed to validate Ion Torrent data. PCR of D-loop was performed using forward (5’TCAAGGAAGAAGCAATAGCC 3’) and reverse (5’GGATTGTTGGGCGTGTAAA 3’) primers. Further sequencing was carried out using PCR primers and internal primer (5’TATTCTCTATGCGGGGGTTC 3’) using Big Dye v3.1 Chemistry (Applied Biosystems) on 3500 Genetic Analyzer (Applied Biosystems) following manufacturer’s protocol.

### Data analysis

Reads longer than 100 bp were mapped to the *P*. *pardus* reference mitogenome [GenBank: NC_010641], using CLC Genomic Workbench 5 [32]. Assembled mitogenome was annotated, using MITOS online mitochondrial genome annotation server [14]. Control region repeats RS-2 and RS-3 were identified using Tandem Repeats Finder [33].

### Molecular phylogenetic analysis

Phylogeny of *P*. *leo* was constructed based on partial and full mitochondrial gene cluster (12S rRNA+16S rRNA+ND2+ND4+ND5+ATP8+Cyt b). Partial *P*. *leo* genes having accession number A79300, AF006457, AY170043, AY634398, AF006458, S79302 and DQ899945 [27] were retrieved from NCBI. Full sequences of above same genes of *P*. *leo persica* were compared with gene sequences of *P*. *uncia* [NC_010638], *P*. *pardus* [NC_010641], *P*. *tigris* [NC_010642], *P*. *tigris amoyensis* [NC_014770], *N*. *nebulosa* [NC_008450], *A*. *jubatus* [NC_005212], *P*. *concolor* [NC_016470], *L*. *rufus* [NC_014456], *F*. *catus* [NC_001700] and *P*. *bengalensis euptilurus* [NC_016189] obtained from NCBI organelle genome resource (Table 1). The data was subjected to Maximum likelihood (ML) and Maximum parsimony (MP) methods using MEGA v5.05 software package [34] to establish phylogenetic relationship.

These seven genes were concatenated and multiple alignment was carried out using ClustalW built-in MEGA. The number of bootstrap replicates was set to 1000 and phylogeny was constructed using Hasegawa-Kishino-Yano model, determined by MEGA 5.05 as the best fitting nucleotide substitution model.

### Divergence time estimation

Multiple sequence alignment of genomes was carried out and phylogenetic tree was constructed using HKY+G+T substitution model at 1000 bootstrap replicates. Divergence times were estimated between species using Bayesian evolutionary analysis by sampling trees (BEAST v1.7.1) software [35]. Markov Chain Monte Carlo (MCMC) procedure was used within a Bayesian analysis framework to estimate posterior distributions of evolutionary rates and divergence times. These analyses were performed using DNA sequence alignment of complete mitogenomes, excluding control region. Divergence times were estimated using an uncorrelated lognormal relaxed clock to account for lineage-specific rate heterogeneity as well as using strict molecular clock model (Additional file 1: Figure S1). Bayesian MCMC analyses in BEAST were performed using HKY model of evolution and gamma+ invariant rate heterogeneity models. Divergences were estimated under a Yule speciation process that is generally more appropriate when considering sequences from different species [36]. Monophyletic constraints were imposed for the node to calibrate evolutionary rates. Normal priors were used for the times to the most recent common ancestor (TMRCA) of Pantherinae and Felinae is (mean 10.78 ±1.87 mya), based on the posterior distributions obtained [29].

Simultaneous Markov chains were run for 10,000,000 generations, sampling every 1000 steps. Thus the “.trees” file will contain 10,000 trees and after removing 10% burnin, tree was annotated using TreeAnnotator. Posterior probability limit was set to zero to annotate all nodes. Maximum clade credibility tree was selected for target tree to find highest product of the posterior probability for all nodes. The phylogenetic tree graphic was generated using FigTree v1.3.1 [37].

### GenBank accession number

The complete mitogenome sequence with gene prediction and functional annotation was submitted to GenBank with accession no. KC834784. The raw sequence data generated from Ion Torrent was submitted to SRA with accession no SRR821548. Mitochondrial D-loop sequence was submitted to GenBank with accession no. KC917264.

## Abbreviations

DHU arm: Dihydrouracil arm; HVS: Hypervariable sequence; ML: Maximum likelihood; MP: Maximum parsimony; Mya: Million years ago; NGS: Next generation sequencing.

## Competing interests

The authors declare they have no competing interests.

## Authors’ contributions

AKS, SBB and MNJ conceptualized this study, ASP, OVP, RPP and FPJ carried out the mtDNA extraction, SBB, MNJ, RVP, ASP, AS and RPP performed the sequencing, SBB and SMD carried out the mitogenome assembly, annotations and phylogenetic studies. SBB and team drafted the manuscript. All authors read and approved the final manuscript.

## Supplementary Material

Additional file 1: Figure S1 Phylogenetic tree and divergence time estimates based on strict molecular clock model. Numbers above the nodes represent posterior probabilities. Numbers at the node represent estimated divergence times [FigTree file is also uploaded as “S1FigTree”]. Click here for file
