# Supplementary figures and images for: Complete mitogenome of asiatic lion resolves phylogenetic status within Panthera
Source: BMC Genomics. 2013 Aug 23;14:572. doi: 10.1186/1471-2164-14-572 (PMC3765570; doi:10.1186/1471-2164-14-572)

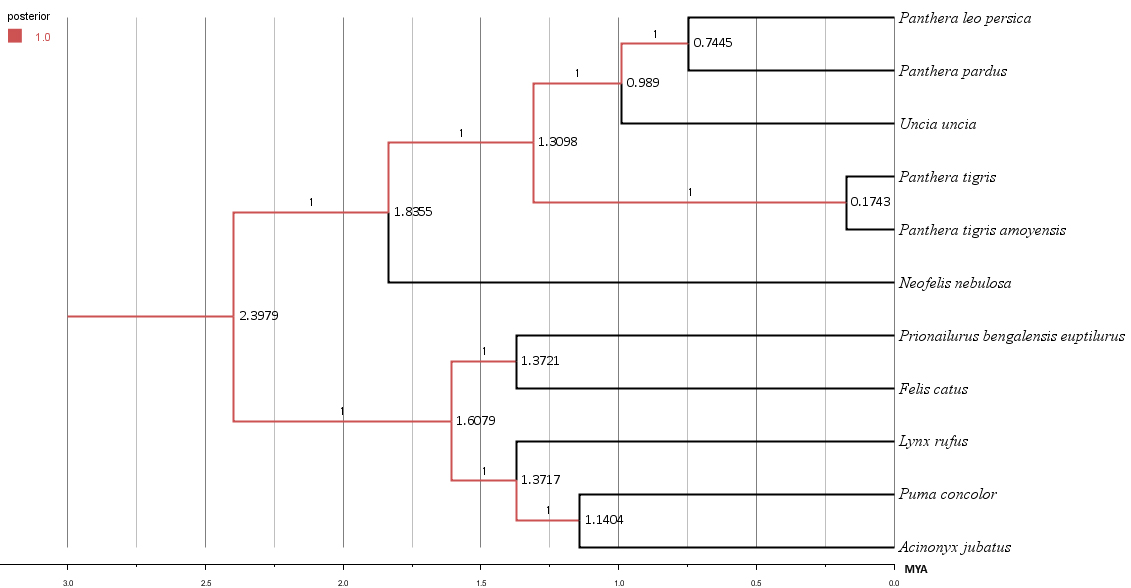

Supplement: Additional file 1: Figure S1 — Phylogenetic tree and divergence time estimates based on strict molecular clock model. Numbers above the nodes represent posterior probabilities. Numbers at the node represent estimated divergence times [FigTree file is also uploaded as “S1FigTree”]. [file 1471-2164-14-572-S1.jpeg]
